# Employing large language models for emotion detection in psychotherapy transcripts

**DOI:** 10.3389/fpsyt.2025.1504306

**Published:** 2025-05-09

**Authors:** Christopher Lalk, Kim Targan, Tobias Steinbrenner, Jana Schaffrath, Steffen Eberhardt, Brian Schwartz, Antonia Vehlen, Wolfgang Lutz, Julian Rubel

**Affiliations:** ^1^ Department of Psychology, Osnabrück University, Osnabrück, Germany; ^2^ Department of Psychology, University of Trier, Trier, Germany

**Keywords:** natural language processing, computational psychotherapy research, machine learning, explainable artificial intelligence, symptom severity, alliance, process-outcome-research

## Abstract

**Purpose:**

In the context of psychotherapy, emotions play an important role both through their association with symptom severity, as well as their effects on the therapeutic relationship. In this analysis, we aim to train a large language model (LLM) for the detection of emotions in German speech. We want to apply this model on a corpus of psychotherapy transcripts to predict symptom severity and alliance aiming to identify the most important emotions for the prediction of symptom severity and therapeutic alliance.

**Methods:**

We employed a public labeled dataset of 28 emotions and translated the dataset into German. A pre-trained LLM was then fine-tuned on this dataset for emotion classification. We applied the fine-tuned model to a dataset containing 553 psychotherapy sessions of 124 patients. Using machine learning (ML) and explainable artificial intelligence (AI), we predicted symptom severity and alliance by the detected emotions.

**Results:**

Our fine-tuned model achieved modest classification performance (*F1_macro_* =0.45, *Accuracy*=0.41, *Kappa*=0.42) across the 28 emotions. Incorporating all emotions, our ML model showed satisfying performance for the prediction of symptom severity (*r* = .50; 95%-CI:.42,.57) and moderate performance for the prediction of alliance scores (*r* = .20; 95%-CI:.06,.32). The most important emotions for the prediction of symptom severity were *approval, anger*, and *fear*. The most important emotions for the prediction of alliance were *curiosity, confusion*, and *surprise*.

**Conclusions:**

Even though the classification results were only moderate, our model achieved a good performance especially for prediction of symptom severity. The results confirm the role of negative emotions in the prediction of symptom severity, while they also highlight the role of positive emotions in fostering a good alliance. Future directions entail the improvement of the labeled dataset, especially with regards to domain-specificity and incorporating context information. Additionally, other modalities and Natural Language Processsing (NLP)-based alliance assessment could be integrated.

## Introduction

Emotions can be conceptualized as “biologically based reactions that coordinate adaptive responding to important opportunities and challenges” (1, p. 152). There are qualitative differences between emotions based on the opportunity or challenge that the emotion has been evolved to address, as well as its expression (2). Further, emotions can immediately impact behavior, quickly regulating one’s behavior based on the situation (3). For instance, anger may be an adaptive reaction to an unfair treatment (4), prompting the person at which it is targeted to change their behavior according to the demand. Simultaneously, anger comes with specific facial features (5) and associated behaviors, such as speaking forcefully or even yelling.

Since emotions are an essential part of daily functioning, it is important that individuals are able to regulate them, that is, modulate the emotional experience and its expression (1). People who lack this ability, may be compromised in several domains of life, which could negatively affect mental health (1, 6). Therefore, it comes as no surprise that many mental health disorders are associated with emotion regulation deficits (7).

Because of the importance of emotion regulation processes across various mental health disorders, emotions play an essential role in psychotherapy (8). Most importantly, they are associated with symptom severity in mood and anxiety disorders (9–11). Even though, emotions and affect can be differentiated (e.g., affect is longer in duration, is less intentional, tends to have unknown causes, and has lower intensity), there is substantial overlap between both constructs (9). For instance, the correlation between fear (emotion) and anxiety (affect) was calculated as *r* = .72, amounting to more than 50% of shared variance (10). Therefore, the measurement of someone’s emotions can provide an estimate of their affect. Simultaneously, dysregulated affect is an important feature in anxiety and depression (11), which are characterized by excessive negative (NA) and a lack of positive affect (PA). Generally, affect is sensitive to change: In a meta-analysis, psychotherapy for depression has been shown to decrease NA and increase PA (12).

According to the broaden-and-built theory, PA helps to strengthen and build resources by extending one’s thought-action repertoire (13). For instance, PA comes with the urge to play, explore, savor, and connect, all of which can foster resources through the creation of new opportunities and strengthening of relationships. This, in turn, raises wellbeing and PA (13). Consequently, it is not surprising that PA can protect against depression (14), may mediate depression recovery (15), and that lack of PA is associated with typical depression symptoms, such as sadness, loss of interest, little energy, and apathy (16, 17). Conversely, NA is linked with stress levels (18) and depressive symptoms (19). NA is a better predictor of depression deterioration than PA, differentiates better between depressed and healthy individuals (20), and predicts future depression onset while PA does not (21). Therefore, NA is likely a better marker for symptom severity than PA.

Beyond their association with symptom severity, emotions play another crucial role in the context of psychotherapy through their impact on the therapeutic alliance, which is one of the best predictors of successful treatment (22). Human beings are fundamentally social creatures and emotions are an essential mechanism for the regulation of social relationships (23). Much of the adaptive strength of emotions is mediated by their effects on interpersonal functioning so that they can be described as intrinsically interpersonal (24): Emotion expression helps people to recognize our needs and wishes, allowing them to support us and strengthening the mutual bond. However, positive interpersonal effects of emotion expression are not automatically given, but depend on several crucial factors (23). In particular, emotion regulation plays an important role: For instance, individuals with high levels of NA report more difficult and less satisfying romantic relationships (25). Though negative emotions can play an adaptive role as well, the excessive expression of NA can be devastating for romantic relationships, because it perpetuates a reciprocal spiral, from which it is difficult to disengage (26). Similarly, the expression of negative sentiment towards the therapist or the therapeutic situation is associated with lower levels of alliance (27, 28). Depressed mood is associated with lower levels of emotion regulation, which can lead to anger management difficulties, reduced trust and forgiveness, heightened levels of social comparison, as well as social withdrawal, all of which can impair social functioning and therefore harm the therapeutic relationship (29). In summary, patient emotions associated with either withdrawal or confrontation and criticism may be particular harmful to the alliance (30). For example, patients with a lot of shame tend to withdraw, which leads to negative effects on the alliance (31), while anger, hostility, and frustration can impair the alliance via confrontation and criticism (27, 28).

Contrary, positive emotions serve important social functions, which can improve the therapeutic alliance (32–35). Most importantly, they can increase intimacy and emotional bond, as well as enhance motivation to achieve shared goals (35), both of which are pillars of the working alliance (36). These considerations are confirmed through longitudinal studies, that have shown bidirectional effects between positive emotions and alliance (32, 34). Though we did not find results regarding the relative strength of negative and positive emotions on the therapeutic relationship, it is likely that negative emotions may have the greater impact, since negative events tend to have greater effects on most areas of life, including interpersonal relations (37).

Emotions can be assessed via many different means, including video, audio, electroencephalography (for an overview see 38), electromyography, various other physiological measures (e.g., heart rate, blood pressure), or a combination of several modalities (39–41). Different methods have been successfully employed depending on the data source. For electroencephalography (see 38), features can be employed for machine learning from different domains, such as the time, frequency, or both. Additionally, employing deep learning, the raw data can be used without feature engineering. For instance, using frequency features, good accuracy (>80%) has been achieved both for the classification of valence and arousal (42). Similar approaches are possible for other physiological data (39). For video emotion classification, deep learning models show competitive performance with a convolutional neural network achieving 66% accuracy in a facial classification (43). In the voice domain, a combination of convolutional neural networks and a transformer architectures (wav2vec 2.0; 44) shows state-of-the-art performance across various tasks (45). For text data, current models successfully employ a transformer architecture (e.g., 46).

However, better results can be achieved for multimodal models. For instance, automatic classification of basic emotions based on text, speech, and video in a hidden Markov model achieved good accuracy in an experimental setting (47). Regarding a naturalistic psychotherapy setting, emotions were mainly assessed via questionnaire measures (48) with notable exceptions, where emotions were judged by human raters (e.g., 49, 50). However, both of these approaches have drawbacks: Questionnaire measures can be burdensome for patients and are unable to track emotions over the course of a session. While human raters can indeed track emotions over the session, this is very time-consuming, so that it is difficult to apply to large session datasets. With the emergence of artificial intelligence (AI) and natural language processing (NLP), new approaches for the automatic analyses of large language corpora are available (51). NLP has already shown promising success in the identification of therapist skills (52), motivational interview adherence (53), or relevant session themes (54, 55). In the context of emotion detection, M. Tanana et al. (56) trained uni-, bi-, and trigram^1^ models on the detection of sentiment on 100,000 rated utterances from psychotherapy transcripts. This work was later extended by the inclusion of the transformer model Bidirectional Encoder Representations and Transformations (BERT; 57) and a model based on positive and negative affective words from the Linguistic Inquiry and Word Count (LIWC; 58). In this analysis, the BERT model showed the best performance (59). More recently, Eberhardt et al. (60) validated the performance of another transformer model on a set of 85 transcripts. They found significant correlations between automatically calculated sentiment and patient- and therapist-reported emotions. Further, symptom severity was significantly associated with negative sentiment.

While these results provide evidence for the reliability and validity of sentiment analysis, they are restricted to the valence dimension, classifying all utterance on a single dimension from negative to positive. Though the valence dimension is highly relevant in this context, at least six basic emotions can be distinguished with additional affect states (2), that can be organized across multiple dimensions (e.g., valence, intensity, intentionality, duration) and multiple categories (e.g., causes, function, mimic, behavior).

### Objectives

Therefore, we aim to fine-tune a large language model (LLM) for a more fine-grained analysis of emotions in the German language. We want to show the clinical utility of this approach by applying this model to a dataset of psychotherapy sessions to predict symptom severity and alliance, employing explainable AI to identify the most important emotions for the prediction of both.

### Hypotheses

We expect our fine-tuned German model to accurately capture emotions in the transcript, allowing for a prediction of patient symptom severity. Regarding symptom severity, we expect negative emotions to predict higher symptom severity and positive emotions to predict lower symptom severity. Further, we expect negative emotions to have a higher impact on the prediction of symptom severity than positive emotions. Regarding the alliance, we expect positive emotions to predict better alliance. For negative emotions, we expect lower levels of alliance in general, though particularly for emotions associated with withdrawal (such as embarrassment and confusion) or confrontation (such as anger and disapproval). Again, we expected negative emotions to have a higher impact on the alliance scores.

## Methods

### Patients and therapists

Our dataset contained 124 patients (65.8% female) who had received treatment at an university outpatient clinic in Trier, Germany. On average, patients were 38.8 years (*SD* = 12.7) old. Regarding the socioeconomic status, almost all had either finished a secondary school certificate (51.6%) or their A-levels (42.1%). Most had finished an apprenticeship (41.3%), while 18.2% were currently in training or studying and 11.6% had a university degree. All patients underwent a diagnostic interview employing the Structured Clinical Interview for Axis I DSM-IV Disorders-Patient Edition (SCID-I; 61). They were mostly diagnosed with primary diagnoses of affective disorders (*n* = 56), anxiety disorders (*n* = 24), and trauma and adjustment disorders (*n* = 16). On average, they received 2.3 (*SD* = 1.3, *min* = 1, *max* = 5) comorbid diagnoses.

The treatment was conducted by 47 therapists with a psychology master degree. All therapists had at least one year of prior treatment experience and were either already licensed CBT therapists or currently enrolled in training. They received supervision regularly.

### Treatment

The treatment consisted of weekly CBT sessions. While the first two sessions served diagnostic purposes (initial assessment in session 1 and SCID-I interview in session 2), the treatment began in the third session. On average, patients received 35.7 (*SD* = 19.7) sessions.

### Instruments and measures

#### Symptom severity

Prior to each session, symptom severity was assessed via the Hopkins Symptom Checklist-11 (HSCL-11; 62). The HSCL-11 is an 11-item self-report scale about general psychological distress. Patients rated a list of 11 symptoms (fearfulness, anxiousness, agitation, panic, sleep problems, hopelessness, loneliness, low mood, lack of interest, suicidal ideation, and worthlessness) on a Likert-type scale from 1 (*not at all*) to 4 (*extremely*). Symptom severity was then calculated as the mean score on these items. The HSCL-11 contains a depression and anxiety subscale and is highly correlated with various other anxiety and depression questionnaires (63). For instance, high associations have been found for the Brief Symptom Inventory (BSI; 64; *r* = .91) and its subscales for anxiety (*r* = .82) and depression (*r* = .91). Regarding depression, high correlations have also been found for the Patient Health Questionnaire-9 (PHQ-9; 65; *r* = .81). For worry symptoms, high correlations exist with the Generalized Anxiety Disorder 7 (GAD-7; 66; *r* = .72). The HSCL-11 has shown good sensitivity to change (67). Assessing the third session of each patient in this sample, we also found good internal consistency (ω = .92) according to McDonald’s omega (68). Across all sessions in our dataset, average symptom severity was at 2.2 (*SD* = 0.79) on a scale from 1 to 4.

#### Alliance

Therapeutic alliance was assessed by the patient via the Session Rating Scale (SRS; 69). The patients filled the questionnaire after each session. The SRS contains the conceptualization by Bordin (36) of three alliance components: 1. Affective bond, 2. Goal agreement, 3. Task agreement, which are assessed by three items. The SRS contains an additional fourth item, which reflects the overall alliance. The final score is calculated as a mean across all four items. The SRS has shown generally satisfying reliability, ranging from *α* = .70 to.97 and a test-retest reliability between *r* = .54 and.70 (70). In our sample, the SRS showed a good internal consistency in the third session (*ω* = .83). Correlations with other alliance measures have been moderate (HAQ-II; *r* = .48; 69; WAI; *r* = .57 –.65; 71).

### Transcripts

The transcripts corpus consisted of 553 transcripts of psychotherapy sessions. On average, there were 4.5 (*SD* = 4.9) transcripts per patient, generally starting with session 3 and continuing with every fifth session (e.g., 3, 5, 10, …).

Transcription was conducted without the use of transcription software by psychology students based on the session recordings. Names of persons or places were removed to reduce identifiable information. The transcripts contained sparse annotations about nonverbal behavior or interruption in parentheses. Each transcript was organized as a table of consecutive speech turns by therapist and patient. The transcripts were not labelled for emotions. For our analysis, we retained only the patients’ speech turns, leaving 104,557 speech turns. We further split these speech turns into sentences for the model inference and retained all sentences with at least three words, leaving 233,648 sentences. Average sentence length was 12.7 (*SD* = 10.4) words. Per session, patients spoke on average 422.5 (*SD* = 174.1) sentences, amounting to 5,362.6 (*SD* = 2,089.3) words.

### GoEmotions dataset

**Figure 1:**
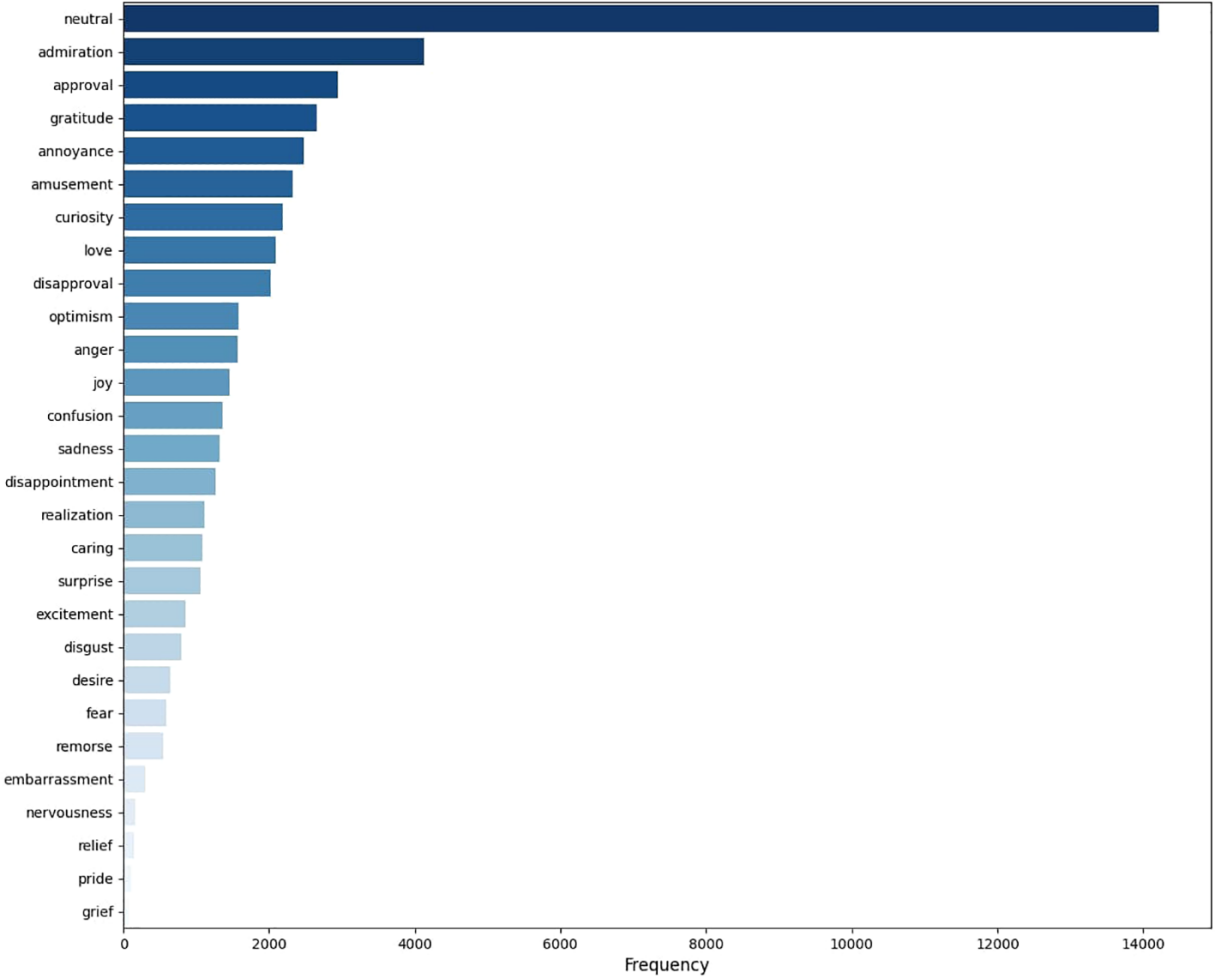
Barplot of the emotion frequency in the train set of GoEmotions.

We used the *GoEmotions* dataset (72) as our labeled training data. The dataset consists of 54k labeled comments. All comments were taken from Reddit, while excluding offensive, vulgar, religion, and identity words. Comments had a length between 3 and 30 words and were balanced across the sentiment, different emotions, and subreddits. The comments were annotated by three raters across a list of 28 emotions (or respectively 27 emotions and a ‘neutral’ category). If reasonable, multiple labels could be given to a single comment. Interrater agreement was assessed via Cohen’s kappa by Demszky et al. (72), ranging between 0.331 (*grief*) and 0.468 (*admiration*). As is shown in **Figure 1**, the posts were not well balanced across all emotions with especially neutral and admiration and approval being the most prevalent ones, while relief, pride and grief were very rare.

### XLM-RoBERTa-base language model

We employed the *XLM-RoBERTa-base* model (73) as our base LLM, which we fine-tuned on the dataset. The *XLM-RoBERTa-base* model was trained on a masked multilingual dataset of over 2 terabyte (of which 66.6 gigabyte were in German), which allows the model to perform in over 100 languages. The model has an embedding size of 1024 tokens, corresponding to a maximal input context length of about 600–800 words. The *XLM-RoBERTa-base* model was pretrained with a Masked Language Modeling objective by predicting 15% of randomly masked words to learn a bidirectional representation of the sentence. Since it was only trained on raw texts without human labeling, it is intended to be fine-tuned on a downstream task, such as the classification task that is contained in this paper.

### Data analytic strategy

**Figure 2:**
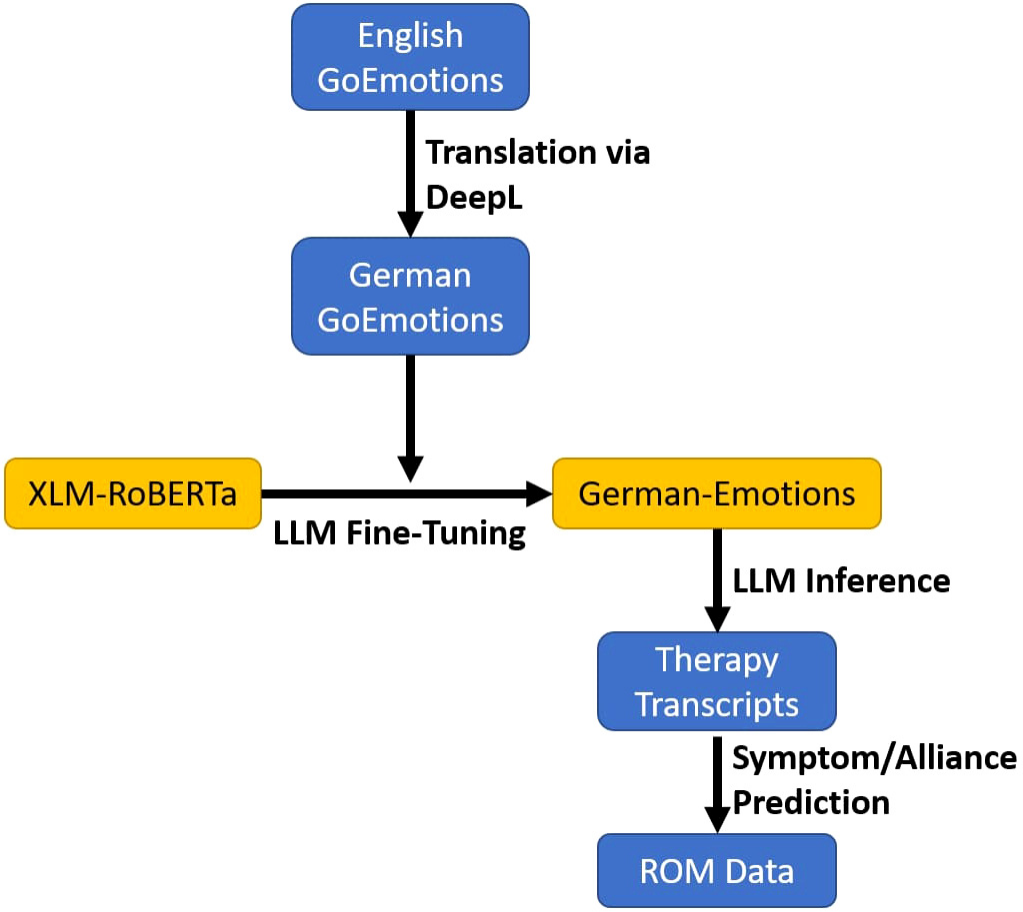
Complete workflow for the data analysis (blue represents data and yellow the LLMs). The GoEmotions dataset is translated into German and used to fine-tune the XLM-RoBERTa LLM. The LLM is then employed to infer the emotions from the therapy transcripts to predict the Routine Outcome Monitoring (ROM) data.

The analyses were conducted with Python 3.9. The complete workflow is shown in **Figure 2** and is elaborated below.

#### Pre-processing and large language model fine-tuning

We selected the 54k labeled comments from the raw *GoEmotions* dataset and used automatic translation to German via DeepL (74). The dataset was split into 80% training set, 10% validation set, and 10% test set. Emotions were classified to a comment via one-hot encoding^2^. We then fine-tuned the *XLM-RoBERTa-base* model on the data in a multilabel classification task (i.e., several emotions could be classified to one sentence) with a batch size of 16, learning rate of 3e–5, and weight decay of 0.01. The complete training code can be accessed via the OSF repository (75). Training was conducted for 10 epochs in the dataset format, which allows for faster processing speed (76). The loss function was Binary Cross-Entropy (BCE) with logits because of the multilabel implementation.

**Figure 3:**
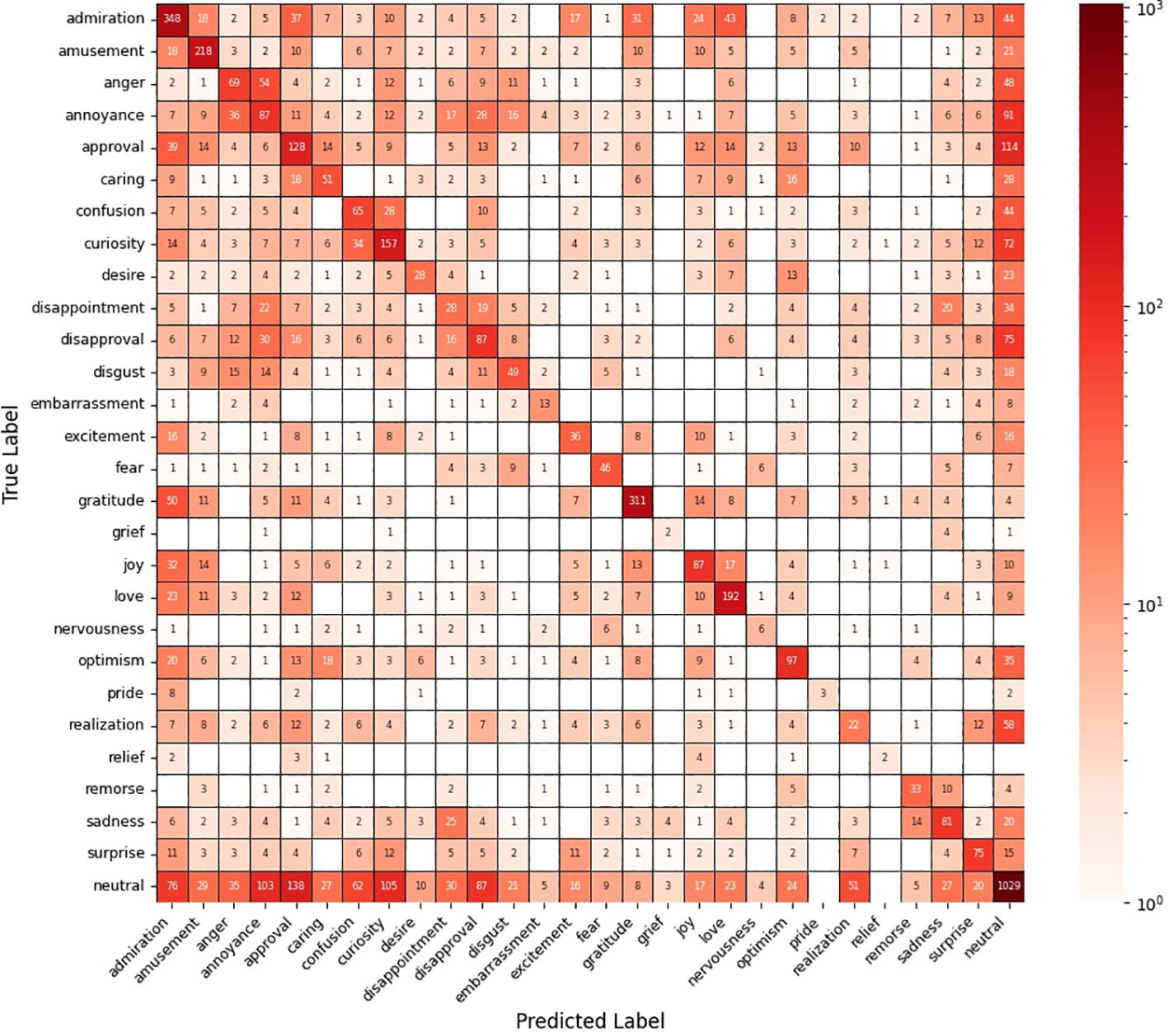
Confusion matrix in the final test set. The color grading is exponentially scaled.

The final model showed similar metrics in the test set (*F1_macro_* = 0.45, *Kappa_macro_* = 0.42, *Accuracy* = 0.41) as the original model (*F1_macro_* = 0.46) by Demszky et al. (72). Cohen’s Kappa calculated between predicted emotions and labeled emotions ranged between 0.15 and 0.88 with a mean of 0.42, indicating moderate agreement (77). The confusion matrix in the final test set is shown in **Figure 3**.

#### Large language model inference

After the fine-tuning was completed, we applied the model for the emotion classification in our dataset. As the labeled data had a length of 3 to 30 words, we decided to conduct the classification on a sentence level. Each sentence by a patient was then run through the model pipeline and was classified across the 28 different emotions by assigning each emotion a probability between 0 and 1 for each sentence. For example, if a sentence was given a probability of 0.8 for *admiration*, this could be interpreted as the predicted probability for the presence of *admiration* in the sentence. Therefore, higher values correspond to a higher probability for the presence of a given emotion.

#### Evaluation strategy for the prediction of outcome

For the prediction of outcome (i.e., alliance and symptom severity), the automatically classified emotion probabilities were aggregated at a session level by calculating the mean for each emotion per session. Since the symptom severity measure contained a depression and an anxiety subscale, we conducted two sensitivity analyses, predicting the respective subscale. The 28 aggregated emotion probabilities were the features for the prediction. Employing nested cross-validation, several machine learning algorithms competed against each other in an internal five-fold cross-validation, while only the best performing algorithm was selected as the algorithm of choice for the respective test set in the external ten-fold cross-validation via the python library XRAI (78). We evaluated the model performance via correlation (*r;* 79), normalized root mean squared error (NRMSE; 80), and mean absolute error (MAE; 81). For the NRMSE, normalization was conducted by dividing through the standard deviation of the target variable. Confidence intervals were calculated by bootstrapping across the ten test folds.

#### Machine learning algorithms

In order to achieve good prediction metrics, we chose a diverse set of ML algorithms to account for feature interactions, nonlinear effects and collinearity. The following machine learning algorithms competed against each other in the internal cross-validation: 1. Least Absolute Shrinkage and Selection Operator (Lasso; 82), 2. Elastic net regularization and variable selection (Elastic Net; 83), 3. eXtreme Gradient Boosting (XGBoost; 84), 4. Random Forest (RF; 85), 5. Support Vector Regression (SVR; 86), and 6. SuperLearner (87). The SuperLearner integrates the ensemble of previous learners (i.e., Lasso, Elastic Net, XGBoost, RF, and SVR), using their predicted scores as features for an SVR meta-learner.

Out of these six different algorithms, the machine learning algorithm with the best mean correlation in the internal five-fold cross-validation was selected as the algorithm for the external test fold. Therefore, it could be possible that different algorithms were selected across the ten test folds, e.g., five times XGBoost, three times RF, and two times SVR.

#### Model explanation

In general, machine-learning models are not very well explainable due to their complex and nonlinear modeling (88). However, one proposed solution has been the use of Shapley values (89), which allow for an estimation of feature impact. To this end, we employed the python package *SHAP* (SHapley Additive exPlanations; 90, 91). *SHAP* allows for the assessment of the individual feature impact (i.e., how much are the predicted values influenced by this feature)?, as well as the impact of groups of features. Further, *SHAP* can be used to assess the direction of a feature impact (e.g., do higher feature values predict higher outcome values)?, as well as interactions between features.

## Results

### Descriptive statistics

The mean probability of all emotions and their reliability is presented in **Table 1**. In addition, the valence of each emotion is indicated (positive/negative/neutral). Altogether, the model classified 14 positive emotions, 12 negative emotions and 2 neutral emotions. The most probable emotions in the transcript corpus were (with the exception of *neutral*) *approval* (12.98%)*, disapproval* (6.11%)*, confusion* (4.32%), and *realization* (4.14%). The least probable emotions were *grief* (0.11%), *remorse* (0.27%), *amusement* (0.73%), and *relief* (0.58%). Regarding the reliability of the assessment, we provided the F1 metric and Cohen’s Kappa (77) from the test set in the *GoEmotions* dataset. F1 is the harmonic mean between precision (how often is the model correct when it predicts the emotion)? and recall (how often does the model detect the emotion when it actually occurs)?. Kappa can be interpreted according to (77) as fair agreement (>.2), moderate agreement (>.4), substantial agreement (>.6), and almost perfect agreement (>.8). Reliability was substantially high for some positive emotions (e.g., *admiration, amusement, gratitude*, and *love*), while the negative emotions showed moderate agreement at best (e.g., *fear*, *remorse*, and *sadness*).

**Table 1:** Mean probability and standard deviations, as well as F1 and kappa values for each emotion.

Emotion (pos./neg./neutral)	M	SD	F1	Cohen’s Kappa
admiration (pos.)	2.35%	1.41%	.64	.601
amusement (pos.)	0.73%	0.66%	.78	.767
anger (neg.)	0.88%	0.67%	.38	.358
annoyance (neg.)	3.17%	1.32%	.27	.229
approval (pos.)	12.98%	4.40%	.34	.293
caring (pos.)	0.79%	0.51%	.38	.365
confusion (neg.)	4.32%	2.06%	.40	.378
curiosity (pos.)	2.10%	1.42%	.51	.486
desire (pos.)	0.58%	0.49%	.39	.387
disappointment (neg.)	2.31%	1.03%	.19	.170
disapproval (neg.)	6.11%	2.02%	.32	.286
disgust (neg.)	0.65%	0.45%	.41	.395
embarrassment (neg.)	0.43%	0.41%	.37	.367
excitement (pos.)	0.87%	0.55%	.35	.339
fear (neg.)	1.23%	1.09%	.59	.584
gratitude (pos.)	0.40%	0.34%	.89	.882
grief (neg.)	0.11%	0.19%	.31	.307
joy (pos.)	1.68%	0.97%	.51	.499
love (pos.)	0.75%	0.69%	.73	.721
nervousness (neg.)	0.91%	0.73%	.28	.276
optimism (pos.)	1.43%	0.72%	.53	.512
pride (pos.)	0.24%	0.28%	.30	.299
realization (pos.)	4.14%	1.66%	.17	.150
relief (pos.)	0.58%	0.36%	.27	.266
remorse (neg.)	0.27%	0.25%	.55	.545
sadness (neg.)	1.92%	1.18%	.50	.488
surprise (neutral)	0.50%	0.40%	.53	.514
neutral (neutral)	62.59%	6.13%	.60	.410

pos*.,* positive; neg*.,* negative*; M*, mean; *SD*, standard deviation. *F1*, harmonic mean between precision (ratio between correct and total predictions of the emotion) and recall (ratio between correct predictions and total occurrence of the emotion); *Cohen’s Kappa*, measure of model-rater-reliability. *Mean* and *SD* values are reported from the transcript corpus, while *F1* and *Cohen’s Kappa* were calculated in the *GoEmotions* dataset.

To give some impression about the labeled statements from psychotherapy transcripts, we compared some patient statements that were classified by the LLM with original comments from the GoEmotions dataset for different emotions (see **Table 2**). Due to confidentiality, the psychotherapy statements are from publicly available transcripts. We have provided a full list containing each emotion in our OSF (75).

**Table 2:** Example phrases from GoEmotions and from psychotherapy transcripts for different emotions.

Label	GoEmotions	Transcript
admiration	*aw, thanks! I appreciate that!*	*Yes, I moved them from one wall to the other and it looks really good.*
anger	Ok, then what the actual f** is your plan?	*He then comes up and tries to open them and I’m so angry I don’t want to talk.*
embarrassment	*Oooooffff. That’s real awkward, but I mean that somehow still ended better than I expected so. Kudos ig??*	*I mean, but I’m so ashamed of it.*
fear	*I am afraid to look, but my morbid curiosity draws me to ask.*	*I said, ‘We’re all scared.’ But to my brother, I said, ‘I’m scared.’*
realization	*It’s like you didn’t even read the comment you’re responding to.*	*I really do notice a difference.*
sadness	*so painful to watch*	*But that was hard to hear too, because when I was told that I was losing a tooth, I just started crying.*

Sample statements were provided by publicly available transcript at alexanderstreet.com.

### Prediction of symptom severity

The machine learning model containing the 28 emotions as features showed a good performance with (*r* = .50 (95%-CI:.42,.57), NRMSE = 0.87 (95%-CI:.83,.91) and MAE = .57 (95%-CI:.55,.59). The selected learners for the external cross-validation were RF (6x) and SVR (4x). The most important predictors (see **Table 3**) as calculated according to the relative *SHAP* value were *approval* (9.2%), *sadness* (8.7%), *fear* (7.8%), *disappointment* (6.4%), *desire* (6.3%), and *sadness* (6.2%). We expected negative emotions to predict higher symptom severity and positive emotions to predict lower symptom severity. This was generally accurate, though some positive emotions were associated with higher symptom severity, namely *desire, pride, caring, amusement*, and *love.* Simultaneously, no negative emotion was associated with less symptom severity. Further, we expected negative emotions to have a stronger impact on symptom severity than positive emotions. Altogether, *approval, admiration, optimism, realization, excitement, gratitude*, and *relief* were positive emotions significantly associated with lower symptom severity, pertaining to an aggregated feature impact of 27.0%, while all negative emotions *(anger, fear, disappointment, sadness, nervousness, disapproval, annoyance, embarrassment, confusion, disgust, grief, and remorse)* were significantly associated with higher symptom severity with almost twice the aggregated impact of negative emotions (51.8%), confirming the hypothesis. The remaining emotions were not significantly associated with symptom severity.

**Table 3:** Symptom severity prediction: emotions, relative SHAP value, and correlation with SHAP value.

Emotion	Symptom Severity	
	Relative SHAP value	Correlation with SHAP value (95%-CI)
approval	9.16%	-.63 (-.69, -.55)
anger	8.67%	.86 (.81,.91)
fear	7.77%	.88 (.84,.92)
disappointment	6.42%	.79 (.73,.84)
desire	6.32%	.90 (.85,.95)
sadness	6.24%	.83 (.78,.88)
admiration	5.96%	-.71 (-.79, -.64)
nervousness	4.72%	.79 (.72,.85)
disapproval	3.76%	.82 (.78,.86)
neutral	3.46%	-.01 (-.38,.37)
annoyance	3.38%	.89 (.84,.93)
optimism	3.29%	-.78 (-.83, -.73)
realization	3.18%	-.89 (-.91, -.86)
embarrassment	2.54%	.75 (.67,.83)
confusion	2.43%	.81 (.73,.87)
excitement	2.42%	-.56 (-.65, -.47)
disgust	2.12%	.80 (.75,.85)
pride	2.08%	.73 (.65,.81)
grief	1.98%	.55 (.44,.67)
caring	1.92%	.29 (.04,.51)
surprise	1.90%	-.60 (-.79, -.37)
remorse	1.78%	.33 (.04,.57)
joy	1.62%	-.18 (-.40,.02)
gratitude	1.60%	-.47 (-.70, -.22)
amusement	1.40%	.33 (.12,.53)
relief	1.38%	-.51 (-.65, -.37)
curiosity	1.34%	.14 (-.08,.35)
love	1.14%	.39 (.20,.55)

### Sensitivity analyses for the prediction of the anxiety and the depression subscale

Regarding the prediction of the anxiety (*r* = .47, 95%-CI: .40, .55) and depression (.53, 95%-CI: .47, .59) subscales, good metrics were achieved. A list of all associated emotions and their relative impact can be obtained from **Supplementary Tables 1, 2**. For the anxiety subscale, the emotions with the highest positive impact were *fear* (17.3%), *sadness* (8.6%), and *nervousness* (7.3%), while *approval* (10.4%) and *admiration* (6.1%) had the highest negative impact. Regarding depression, *sadness* (18.6%), *grief* (8.0%) and *disappointment* (7.2%) had the highest positive and *approval* (17.7%) and *realization* (4.2%) the highest negative impact.

### Prediction of alliance

For the alliance prediction, the model performance was low to moderate with (*r* = .20 (95%-CI:´.06,.32), NRMSE = 0.98 (95%-CI:.95, 1.01) and MAE = 8.76 (95%-CI: 8.34, 9.22). Regarding the external test set learners, Elastic Net (4x), as well as Lasso (3x) and RF (3x) were selected. The emotions with the highest impact (see **Table 4**) were *curiosity* (24.9%), *confusion* (16.4%), and *surprise* (5.4%). We expected positive emotions to predict higher alliance and negative emotions to predict lower alliance. For negative emotions, this was not the case in general, since *annoyance, remorse, disgust, nervousness, sadness*, and *embarrassment* were not associated with lower alliance. Though, *confusion* as a marker of a withdrawal rupture and *anger* and *disapproval* as markers of confrontation ruptures predicted lower alliance. Positive emotions were mostly associated with higher alliance with the notable exception of *curiosity* and *approval*. Some emotions (e.g., *optimism, nervousness*) were not significantly associated with alliance. We expected negative emotions (*confusion, anger, disapproval, fear, disappointment, grief;* 27.5%) to have a greater negative impact on alliance than the positive impact of positive emotions (*desire, joy, admiration, excitement, realization, amusement, relief, love, caring;* 28.95%), which was not the case.

**Table 4:** Alliance prediction: emotions, relative SHAP value, and correlation with SHAP value.

Emotion	Alliance	
	Relative SHAP value	Correlation with SHAP value (95%-CI)
curiosity	24.94%	-.96 (-1.00, -.92)
confusion	16.41%	-.93 (-1.00, -.85)
surprise	5.42%	.77 (.55,.95)
desire	4.72%	.84 (.65,.98)
joy	4.69%	.61 (.35,.85)
admiration	4.61%	.56 (.30,.81)
excitement	4.35%	.77 (.49,.98)
realization	4.02%	.65 (.36,.90)
anger	3.85%	-.37 (-.69, -.04)
disapproval	3.36%	-.35 (-.65, -.05)
amusement	3.02%	.62 (.35,.86)
annoyance	2.08%	.49 (.22,.78)
remorse	1.94%	.50 (.15,.80)
approval	1.62%	-.23 (-.46, -.05)
relief	1.57%	.48 (.20,.77)
optimism	1.43%	.05 (-.11,.28)
fear	1.40%	-.31 (-.55, -.08)
disappointment	1.34%	-.53 (-.77, -.25)
grief	1.08%	-.06 (-.31,.25)
neutral	1.08%	.16 (.01,.37)
love	1.07%	.44 (.15,.73)
disgust	1.06%	.40 (.15,.66)
nervousness	1.02%	.01 (-.21,.25)
caring	0.91%	.53 (.26,.80)
gratitude	0.88%	.06 (-.13,.30)
sadness	0.88%	.31 (.07,.61)
pride	0.82%	.07 (-.13,.31)
embarrassment	0.42%	.12 (-.03,.33)

## Discussion

Our study served three purposes: First, we fine-tuned an LLM for the classification of 28 emotions in German. Second, we employed this model on a dataset of 553 psychotherapy transcripts to predict symptom severity and alliance. Third, we assessed the most important emotions for the predictions of symptom severity and alliance. Our results indicate a modest classification performance for our fine-tuned model with *F1_macro_* = .45, which is almost identical to the original performance in English (*F1_macro_* = .46), indicating no overall accuracy loss due to the translation. Looking at individual emotions, classification accuracy varied slightly, but was mostly similar between German and English (the largest decrease in *F1_macro_* was .11). The Kappa value of .42 demonstrated moderate agreement, which is not surprising because of the inherent limitation of identifying emotions only via the transcript modality while ignoring other modalities such as voice intonation (audio; e.g., 92) or facial expression (video; e.g., 93). Therefore, the modest performance likely did result from the low inter-rater-reliability in the *GoEmotions* dataset (Cohen’s Kappa ranging from .33 to .47). However, there was considerable variation regarding the model’s Kappa values, ranging between .15 and .88. This seems logical since some emotions may be more clearly expressed via language content (e.g., *admiration, amusement, fear*) than others (e.g., *disappointment, annoyance*), which might be better captured via voice features or mimic.

Employing the fine-tuned model, we successfully predicted symptom severity (*r* = .50) and alliance (*r* = .20) from the transcripts. The accuracy was comparable to a different approach based on session content as operationalized by topic modeling of 250 topics (55). Emotions showed higher associations with symptom severity than a model based on 14 cognitive distortions (*r* = .33) or negative sentiment only *r* = .08, 94). In contrast, Eberhardt et al. (60) found both within-person correlations for positive sentiment (*r* = −.29) and large between-person correlations for negative sentiment (*r* = .66). However, these correlations must be interpreted with caution as they come from a small dataset (*N* = 79) and may not be stable (95).

Regarding symptom severity, negative emotions mainly predicted higher and positive emotions lower symptom severity, as expected. For negative emotions, the best predictors were *anger* and *fear*, which both come with high arousal. *Anger* may be associated with the HSCL-11 items agitation, low mood and feeling worthless (as it often was self-directed) while *fear* may be associated with various items of the HSCL-11, such as fearfulness, anxiousness, agitation, panic, and sleep problems. Surprisingly, the positive emotion a*pproval* had the highest effect, probably because it was simultaneously the most often detected emotion (13.0%). Though content-wise *approval* was a fuzzy concept with low reliability (*Kappa* = .29), it tended to be associated with statements of agreement, feasibility, and acceptance, which can be protective factors (14, 96). *Desire* was associated with higher symptom severity, likely because it contained statements that indicated a present frustration or lack of something (e.g., desiring more sleep, a more accepting partner, or happiness). Regarding the sensitivity analysis for anxiety, we found the highest impact of the negative emotions *fear, sadness*, and *nervousness*, while *approval* was the positive emotion with the highest impact. For depression, *approval* also was the most important positive emotion, while *sadness, grief* and *disappointment* were the most important negative emotions.

For alliance, contrary to our expectations, only few negative emotions predicted lower alliance, while most positive emotions predicted higher alliance. However, as expected, the negative emotions with significant associations might have served as withdrawal (*confusion*) or confrontation markers (*anger, disapproval*). The negative association with *curiosity* was surprising, especially since it explained about a quarter of the model impact. Looking at the sentences that were classified as *curiosity*, it seemed as if they sometimes indicated a misunderstanding (e.g., “*So this is from my diagnosis*?”), frustration (“*And you’re a psychologist, or what?”*), or confusion (“*What will happen to me then*?”). The positive impact of positive emotions was highlighted by the associations with *desire, joy, admiration*, and *excitement* in line with the literature (35).

## Limitations and future directions

This analysis comes with several limitations. Regarding our transcript-based approach, the training data is not optimal, as it is based on a social media dataset containing comments with a length of only 3 to 30 words. As can be seen from **Table 2**, the social media comments tended to be more informal and contained some slang. Also, the comments seemed more expressive and more intense, which could cause problems with the emotion detection. It would therefore be desirable to train models on labeled data from the relevant domain (i.e., therapy transcripts), incorporating longer and more complex statements, because domain specificity can improve model accuracy (56, 97). Also, it would be desirable to have training set, which is better balanced across emotions, because some emotions (e.g., *grief, pride, relief, nervousness, embarrassment*; see **Figure 1**) hardly occur in the training data. This could lead the model to be biased against these emotions so that they may not be detected. In general, emotion detection could be improved by including other modalities, such as voice (92) or mimic via video (93) due to the superiority of a multimodal approach (98). Altogether, these approaches would likely improve the classification accuracy substantially.

Since some of the emotions (e.g., *realization, approval*) were only loosely connected to the psychological concept of emotions (2), future analyses could benefit from a clearer and more distinct definition of the included emotions. Further, clearly, the relationship between patient emotions and alliance is more complex than assumed in our hypotheses. On the one hand, it has been suggested that the effects of emotional arousal on outcome can be moderated by the alliance so that high arousal can be productive as long as there is a sufficiently strong alliance (8). On the other hand, the therapist may actively manage the alliance so that it may be soothing in the context of high arousal and activating and challenging in the context of (too) little arousal (8). Therefore, NLP could be incorporated to model the alliance over the course of a session to assess the dynamic interplay between emotions and alliance. This could be employed to give feedback to therapists after a session, highlighting moments where they could modulate the interaction to increase or decrease arousal. Another approach would be the integration of therapist emotions and therapist empathy into our model to assess which therapist emotions may be detrimental to the alliance.

## Conclusion

In summary, our transcript-based model could classify 28 emotions with reasonable model-rater-agreement (*F1* = .45, *Kappa* = .42). Classification tended to be more reliable for positive emotions. The average detection probability ranged between 13% (*approval*) and 0.1% (*grief*). Substantial associations with symptom severity (*r* = .50) were found in the expected directions (i.e., positive emotions were associated with lower symptom severity and negative emotions with higher symptom severity) by selecting SVR and RF learners. Associations with alliance (*r* = .20) were lower and the selected learners were Lasso, Elastic Net, and RF. While many positive emotions predicted higher alliance, the results for negative emotions were mixed. Notably, negative emotions that could serve as withdrawal (*confusion;* 16% impact) or confrontation ruptures (*anger, disapproval;* together 7% impact) predicted lower alliance. In the future, this model opens a wide array of possibilities regarding process-outcome associations with emotional processes, such as analyzing the emotion dynamic over the course of a session or comparing patient with therapist emotions.

## Data Availability

The original contributions presented in the study are publicly available. Our fine-tuned LLM "German-Emotions", including some example code, is available via huggingface (https://huggingface.co/ChrisLalk/German-Emotions). Complete code for model training and model inference is available via OSF (https://doi.org/10.17605/osf.io/AKF3H). Our prediction model XRAI can be accessed via GitHub (https://github.com/PsyRes-Osnabrueck-University/xrai). Our translated dataset can be accessed via OSF, including some labeled example statements (https://doi.org/10.17605/osf.io/AKF3H). The original dataset can be accessed via https://paperswithcode.com/dataset/goemotions.

## Footnotes

1 N-grams refer to sets of *n* consecutive words in a corpus.

2 One-Hot encoding is the transformation of categorial data into binary vectors for each category. Contrary to dummy encoding, there is no reference category.
