# Supplementary material for: Employing large language models for emotion detection in psychotherapy transcripts
Source: Front Psychiatry. 2025 May 9;16:1504306. doi: 10.3389/fpsyt.2025.1504306 (PMC12098529; doi:10.3389/fpsyt.2025.1504306)
Supplement: Supplementary file 1 [file Table1.docx]

**OSM 1 - Anxiety Subscale Prediction: Emotions, Relative SHAP Value, and Correlation With SHAP Value**

| **Emotion** | **Anxiety Subscale** | |
| --- | --- | --- |
|  | **Relative SHAP value** | **Correlation with SHAP value (95%-CI)** |
| Fear | 17.33% | .86 (.81, .91) |
| Approval | 10.43% | -.69 (-.80, -.52) |
| Sadness | 8.61% | .75 (.68, .82) |
| Nervousness | 7.31% | .89 (.86, .91) |
| Admiration | 6.13% | -.48 (-.70, -.23) |
| Realization | 5.92% | -.89 (-.92, -.86) |
| Disgust | 5.15% | .64 (.35, .87) |
| Remorse | 4.23% | .73 (.68, .77) |
| Joy | 4.22% | -.40 (-.57, -.22) |
| Annoyance | 4.02% | .67 (.45, .82) |
| Disapproval | 3.95% | .61 (.35, .80) |
| Neutral | 3.40% | -.42 (-.58, -.26) |
| Optimism | 3.15% | -.52 (-.71, -.31) |
| Surprise | 2.79% | -.59 (-.73, -.43) |
| Gratitude | 2.75% | -.47 (-.71, -.24) |
| Excitement | 2.30% | -.01 (-.31, .25) |
| Love | 2.09% | .41 (.17, .66) |
| Caring | 1.56% | .05 (-.04, .14) |
| Curiosity | 1.31% | -.39 (-.64, -.15) |
| Relief | 0.66% | -.15 (-.35, .01) |
| Pride | 0.61% | .03 (0, .08) |
| Embarrassment | 0.53% | .17 (0, .40) |
| Disappointment | 0.51% | .11 (0, .27) |
| Anger | 0.36% | .09 (0, .26) |
| Amusement | 0.33% | -.07 (-.20, 0) |
| Grief | 0.24% | .03 (0, .08) |
| Fear | 17.33% | .86 (.81, .91) |
| Approval | 10.43% | -.69 (-.80, -.52) |
