# Supplementary material for: Employing large language models for emotion detection in psychotherapy transcripts
Source: Front Psychiatry. 2025 May 9;16:1504306. doi: 10.3389/fpsyt.2025.1504306 (PMC12098529; doi:10.3389/fpsyt.2025.1504306)
Supplement: Supplementary file 2 [file Table2.docx]

**OSM 2 - Depression Subscale Prediction: Emotions, Relative SHAP Value, and Correlation With SHAP Value**

| **Emotion** | **Anxiety Subscale** | |
| --- | --- | --- |
|  | **Relative SHAP value** | **Correlation with SHAP value (95%-CI)** |
| Sadness | 18.56% | .84 (.81, .87) |
| Approval | 17.71% | -.80 (-.83, -.76) |
| Grief | 8.02% | .49 (.45, .53) |
| Disappointment | 7.20% | .82 (.63, .91) |
| Realization | 4.22% | -.87 (-.89, -.85) |
| Disgust | 4.22% | .53 (.26, .80) |
| Gratitude | 4.21% | -.60 (-.68, -.48) |
| Surprise | 3.85% | -.86 (-.89, -.83) |
| Excitement | 3.60% | -.48 (-.57, -.36) |
| Desire | 3.59% | .56 (.32, .75) |
| Neutral | 3.08% | -.68 (-.75, -.60) |
| Disapproval | 2.98% | .49 (.23, .73) |
| Optimism | 2.56% | -.40 (-.52, -.26) |
| Joy | 2.23% | -.27 (-.44, -.10) |
| Love | 2.20% | .68 (.44, .86) |
| Anger | 2.15% | .27 (0, .54) |
| Remorse | 2.05% | .48 (.29, .65) |
| Admiration | 1.62% | -.17 (-.34, -.04) |
| Annoyance | 1.46% | .18 (0, .38) |
| Relief | 1.12% | -.14 (-.34, .05) |
| Fear | 1.09% | .26 (.08, .54) |
| Confusion | 0.67% | .23 (.06, .49) |
| Embarrassment | 0.62% | .11 (0, .24) |
| Nervousness | 0.56% | -.14 (-.28, -.03) |
| Curiosity | 0.27% | .01 (-.13, .14) |
| Pride | 0.15% | -.04 (-.11, 0) |
| Amusement | 0% | 0 (0, 0) |
| Caring | 0% | 0 (0, 0) |
